# Suppressing the impact of the COVID-19 pandemic using controlled testing and isolation

**DOI:** 10.1038/s41598-021-85458-1

**Published:** 2021-03-18

**Authors:** Kobi Cohen, Amir Leshem

**Affiliations:** 1 School of Electrical and Computer Engineering, Ben-Gurion University of the Negev, Beer Sheva, Israel; 2 Faculty of Engineering, Bar-Ilan University, Ramat Gan, Israel

**Keywords:** Epidemiology, Electrical and electronic engineering

## Abstract

The Corona virus disease has significantly affected lives of people around the world. Existing quarantine policies led to large-scale lock-downs because of the slow tracking of the infection paths, and indeed we see new waves of the disease. This can be solved by contact tracing combined with efficient testing policies. Since the number of daily tests is limited, it is crucial to exploit them efficiently to improve the outcome of contact tracing (technological or human-based epidemiological investigations). We develop a controlled testing framework to achieve this goal. The key is to test individuals with high probability of being infected to identify them before symptoms appear. These probabilities are updated based on contact tracing and test results. We demonstrate that the proposed method could reduce the quarantine and morbidity rates compared to existing methods by up to a 50%. The results clearly demonstrate the necessity of accelerating the epidemiological investigations by using technological contact tracing. Furthermore, proper use of the testing capacity using the proposed controlled testing methodology leads to significantly improved results under both small and large testing capacities. We also show that for small new outbreaks controlled testing can prevent the large spread of new waves. Author contributions statement: The authors contributed equally to this work, including conceptualization, analysis, methodology, software, and drafting the work.

## Introduction

The outbreak of the COVID-19 has revealed the widespread effects a pandemic can have on all spheres of life from health, to social life, to the economy^1^. The main thrust of efforts to control the spread is to decrease the reproduction rate to flatten the curve of the total number of infected individuals per day. This is necessary to reduce the load on the health system, although the infection may spread in the population over an extended period of time^2,3^. The most widely implemented response to the exponential growth of the infection has been widespread quarantine and lock-downs^4,5^. While isolating people is an effective tool to decelerate the spread, imposing, repeatedly, a complete quarantine for everyone for a relatively long period of time until the virus is suppressed has negative effects on people’s lives. Moreover, if proper population monitoring is not enforced there may be further waves of the disease. Therefore, early detection of Corona positives is of paramount importance to suppress the spread of COVID-19 as early as possible.

The second component in fighting the pandemic is to devise better testing policy. Today, health systems around the world prioritize the administration of tests to people who have had direct contact with an infected person or who are symptomatic. Some countries, such as South Korea, are implementing extensive resources to test people widely and randomly to detect areas where the pandemic is likely to spread. Nevertheless, these strategies do not exploit resources effectively. First, only testing symptomatic people results in a loss of precious time, during which the individual can infect others before symptoms appear. Furthermore, many people (about $10{-}30\%$) are asymptomatic, and thus cannot be identified using this strategy. In addition, testing the entire population on a daily basis to detect people who remain asymptomatic is impossible because the number of tests that labs can either produce or process is limited. For these reasons, this paper address the prioritization of tests using a feedback methodology. Specifically, the key of the proposed method is to test individuals with a high probability of being infected to identify them before symptoms appear. The probabilities of individuals being infected act as an input to selecting individuals for testing for infection. The individuals with the highest probabilities of being infected are selected. Then, their test results act as a feedback to update the probabilities of other individuals being infected, and so on. For example, when an individual is tested, then the probabilities of his or her neighbors being infected increase or decrease, depending on whether the test result is positive or negative, respectively. Then, the future selection of individuals for testing for infection is updated as well. We will show that this, has three important consequences: Reducing the burden on the health system (i.e., reducing the peak of the number of infected people), reducing the total morbidity (i.e., the total number of severe COVID-19 patients), and reducing the economic and social impact (i.e., the total number of people required to stay in quarantine and the overall time people spend in quarantine).

The mitigation of a pandemic can be achieved using a range of interventions to reduce transmission^6^. Recently, partial lock-down strategies have been suggested, in which half of the population remains under lock-down, where the other half is released, in alternation^7^, or by applying a cyclic schedule of 4 days of work and 10 days of lock-down^8^. However, these strategies lead to more than a $50\%$ loss in economic productivity, which we aim to avoid. These strategies are based on the effectiveness of quarantine, and are simple to analyze. However, they do not consider wide-scale testing, which is available. By tracing and quarantining only those at risk (e.g., those in close contact with an infected individual, depending on the duration of contact and distance), epidemics can be contained without using aggressive lock-downs^9,10^, using surveillance and containment measures for patients^11^, contact tracing^12,13^ and mathematical modelling of the transmission dynamics of the infection^14^.

As reported recently in^15^, increasing the COVID-19 test rate per population would have a significant positive impact on mitigating the pandemic around the world. The missing component in most existing methods is that they do not use COVID-19 tests to identify infected people prior to the occurrence of symptoms. There are a number of recent studies that incorporate tests to mitigating the spread^15^, and provide worst-case analysis of a risk-based selective quarantine^16^. Nevertheless, these methods use passive testing in the sense that post-processing the test results is not used to improve future tests. In a recently published *Science* paper^9^, Ferretti et al. concluded that viral spread could be controlled using contact tracing by testing close contacts of positive cases.

In this paper, we argue that suppressing viral spread can be accomplished much more efficiently by developing a controlled sensing methodology for contact tracing, rather than testing close contacts in a deterministic manner as in^9^. We develop a controlled testing methodology to control the spread of the COVID-19 pandemic based on a large-scale stochastic model. *Controlled sensing*, a.k.a active sensing, is based on classic sequential experimental design theory^17,18^, and has attracted growing attention in recent years in various hypothesis testing and dynamic search problems^19–23^. Controlled sensing policies, have also been used to identify influence in social networks^24^, as well as to learn the dynamics in general networks^25^. The basic idea behind controlled sensing theory when translated to the realm of detecting COVID-19 patients is to use the results obtained from previous tests to update the probabilities of individuals being infected, which improves the future selection process of individuals for testing. Intuitively, as more tests are performed, the learner becomes more certain about the true state of the population, which in turn leads to better choices of people to test. This approach was shown to achieve the asymptotic information theoretic bound in various hypothesis testing problems as the error probability approaches zero (see e.g.,^19,20,26–32^). Using the controlled testing methodology that we develop to control the spread of the COVID-19 pandemic, we report a significant reduction (up to $50\%$) in both quarantine rate as well as morbidity rate in typical settings of COVID-19 parameters as compared to existing studies.

Recent studies considered Susceptible, Infectious, Recovered (SIR)-related models and more general compartmental models^33^ to analyze COVID-19. Cooper et al. developed a SIR-based model to investigate the virus spread within a community^34^. Calafiore et al. developed a modified SIR model to study the COVID-19 contagion in Italy^35^. Wangping et al. extended SIR to prediction of the epidemics in Italy compared with Hunan, China^36^. Faranda and Alberti used Susceptible-Exposed-Infected-Recovered (SEIR) model to model the second wave of COVID-19 infections in France and Italy^37^. Annas et al. used it to analyze the COVID-19 spread in Indonesia^38^. Faranda et al. used SEIR model for estimates of infection counts^39^. Other recent studies can be found in^40–42^ and references therein. These models use differential equations to capture the epidemiological models, by assuming that populations are completely mixed. Interactions and spatial effects between individuals are neglected since the populations are modeled as continuous entities (e.g., mean-field analysis)^43–46^. Thus, these models have limitations in modeling spatial aspects of the epidemic spreading, individual contacts and policies that distinguish between various agents based on the micro behavior of the agents, etc. For this reason, we chose to use a stochastic agent-based model that allows us to model these aspects by microscopically tracking the target individuals. It can also easily incorporate new models for infection probability as well as more elaborated spatial models and individual probabilities of being infected. For example, the model can be used to more accurately exploit the duration of contact provided by contact tracing measures.

## Stochastic model for controlled testing

**Figure 1:**
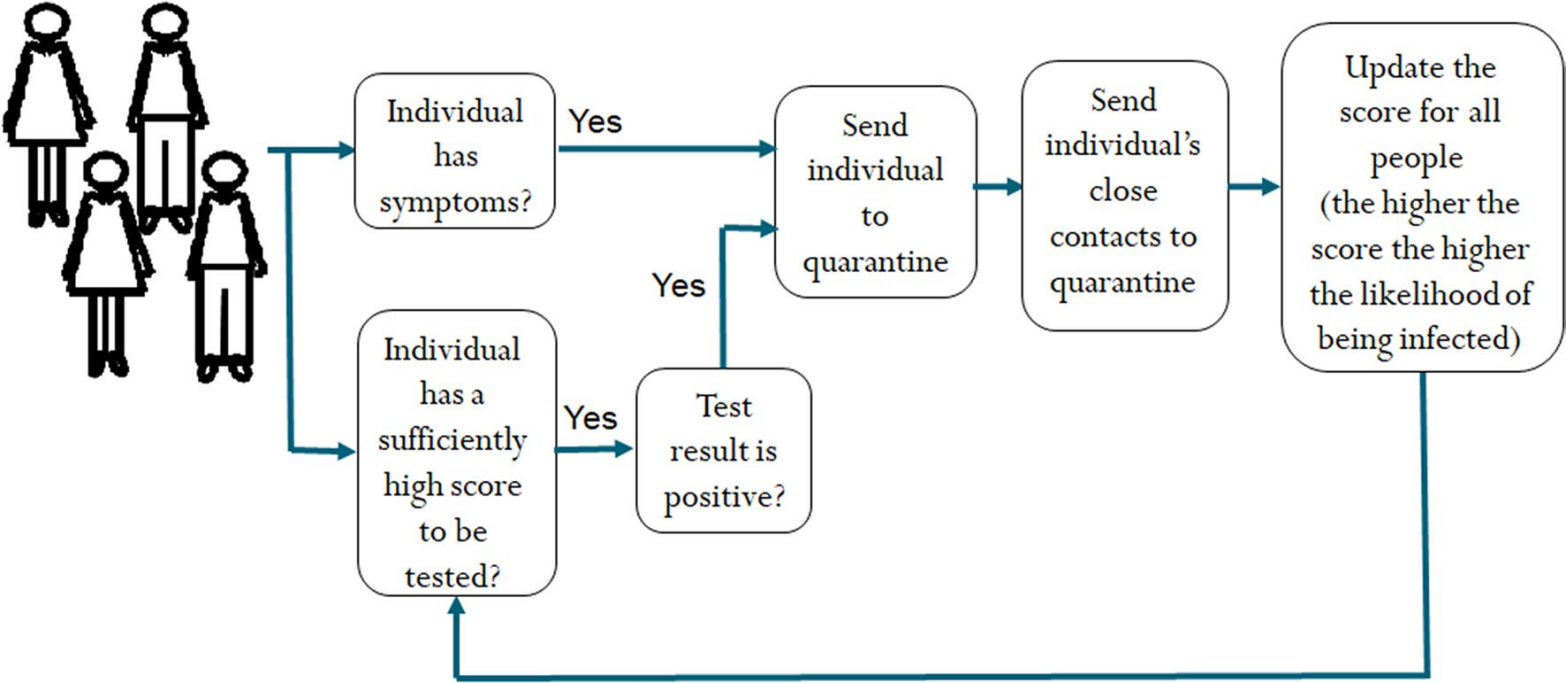
An illustration of the ATI algorithm proposed in this paper.

We start by providing a brief description of the underlying model used in this paper. A detailed mathematical formulation is given in the “Methods” section. The model includes a state for each agent in the population, e.g., healthy, quarantined, infected, recovered, etc. We model the connections between all people as random interactions. For example, being within 1 m of a COVID-19 patient for more than 15 min is defined as close contact by the WHO^4^; and these close contacts are random. Furthermore, we assume that a probability of infection can be assigned to each contact, e.g., using contact tracing devices. The proposed method works as follows: It identifies infected people based on symptoms or test results. Then, people who are declared infected (i.e., have symptoms or get positive test results) and their close contacts (as identified by contact tracing techniques) are quarantined. The outcome of these steps is used to update the score of being infected (i.e., probability) of all people. For example, when an individual is tested, then the probabilities of his or her neighbors being infected increase or decrease, depending on whether the test result is positive or negative, respectively. This update serves as a feedback to the decision on testing where the most likely infected people, i.e., those with highest scores are tested regardless of their symptomatic state. This last step is the feedback that closes the loop seen in Fig. 1. This feedback mechanism is crucial to the success of the proposed method especially given the large proportion of asymptomatic people.

### Description of the active testing and isolation (ATI) algorithm

We now describe the proposed active testing and isolation (ATI) algorithm which controls the testing process. Every day the ATI algorithm works as follows:

1. Identifying infected people and isolating them.
2. Isolating first-order neighbors in quarantine.
3. Updating the belief over the population graph.
4. Actively testing the most likely infected people according to the number of available tests.

An illustration of the ATI algorithm is given in Fig. 1. A detailed description is given in the “Methods” section. Note that the ATI algorithm tests and isolates people actively in a closed-loop manner. Steps 1, 2 identify infected people based on symptoms or test results. Then, people who are declared infected and their first-order neighbors are quarantined. The outcome of these steps is used to update the beliefs of all people in the population graph, which is then used to sample and test the most likely infected people regardless of their symptomatic state. This is the crucial concept that enables ATI to identify infected people early before symptoms appear.

## Results

Our proposed controlled testing technique exploits a large-scale agent-based stochastic model, where we monitor our belief regarding each person’s state and select to test for infection the most probable people to be infected based on belief and the total number of tests available. Beliefs are estimated from contact tracing data as well as symptom reporting. We demonstrate the effectiveness of the technique using a stochastic spread model based on COVD-19 parameters in populations of one million people. We show that we can reduce the quarantine period (defined as the period where more than $1\%$ of the population is quarantined) by up to $50\%$, while reducing both the peak number of infected people per day and the total morbidity by a significant amount. Our methodology allows optimal exploitation of the testing capacity. By testing daily $r_T=0.3\%$ of the population of an infected region, we already show significant benefits. Another important consequence of the results is the significant improvement in performance when shortening the testing and epidemiological investigation period.

We begin by describing simulated results that support our claims. In Table 1, we present the parameters and the value ranges used in the simulations. Then, we provide the details of the proposed active testing and isolation (ATI) (control algorithm) and end with the specifics of the stochastic agent-based model and the related algorithms used to direct the active testing process.

**Table 1:** A list of parameters used in the simulations.

Parameter description	Symbol	Value used in simulations
Number of people (population size)	*N*	$10^5, 10^6$
Ratio of asymptomatic people over the entire population	$r_a$	$30\%$
Initial ratio of infected people over the entire population	$r_i$	0.05–1.5%
Ratio of daily tested people over the entire population (testing capacity)	$r_T$	0.3–5%
Reproduction rate	$R_0$	2.3
Number of days between the day symptoms appear and the day a person and his or her first-order neighbors enter quarantine	$\delta _s$	1–5
Number of days between the day the test is performed and the result is received	$\delta _T$	1–8
Number of days in quarantine for suspected infected people	$D_q$	14
Ratio of disciplined people entering a quarantine out of all people that were ordered to enter a quarantine (quarantine success rate)	$q_s$	70–100%
Number of days between the day of infection and transitioning to the static state (recovering or deceasing)	$D_s$	21
False negatives in test results	FN	0.1–0.2
False positives in test results	FP	0.01

### Performance evaluation

We present three sets of results. The code and data for the simulations can be found in^47^. In the first set we study the possibility of quickly controlling an outbreak in its early phase. This is important for controlling future waves of the disease. Then, in the next two sets, we discuss the amount of testing required to suppress an advanced outbreak with $0.1\%$ of the population already infected. This represents the current state of the disease in many countries. As a baseline for our study we consider three methods: (a) Isolation without testing (NTI) where only symptomatic people are tested. This was the approach, e.g., in Israel umtil mid May 2020; (b) widespread random testing and isolation (RTI), i.e, NTI augmented by widespread tests for anyone interested in getting tested; (c) deterministic testing and isolation (DTI) based on contact tracing as presented in Ferreti et al.^9^. In this technique, when an infected agent is detected, all his or her close contacts are quarantined and tested. The problem here is that when resources are limited it does not necessarily tests the most relevant people. It also incurs a delay in testing second order infections.

**Figure 2:**
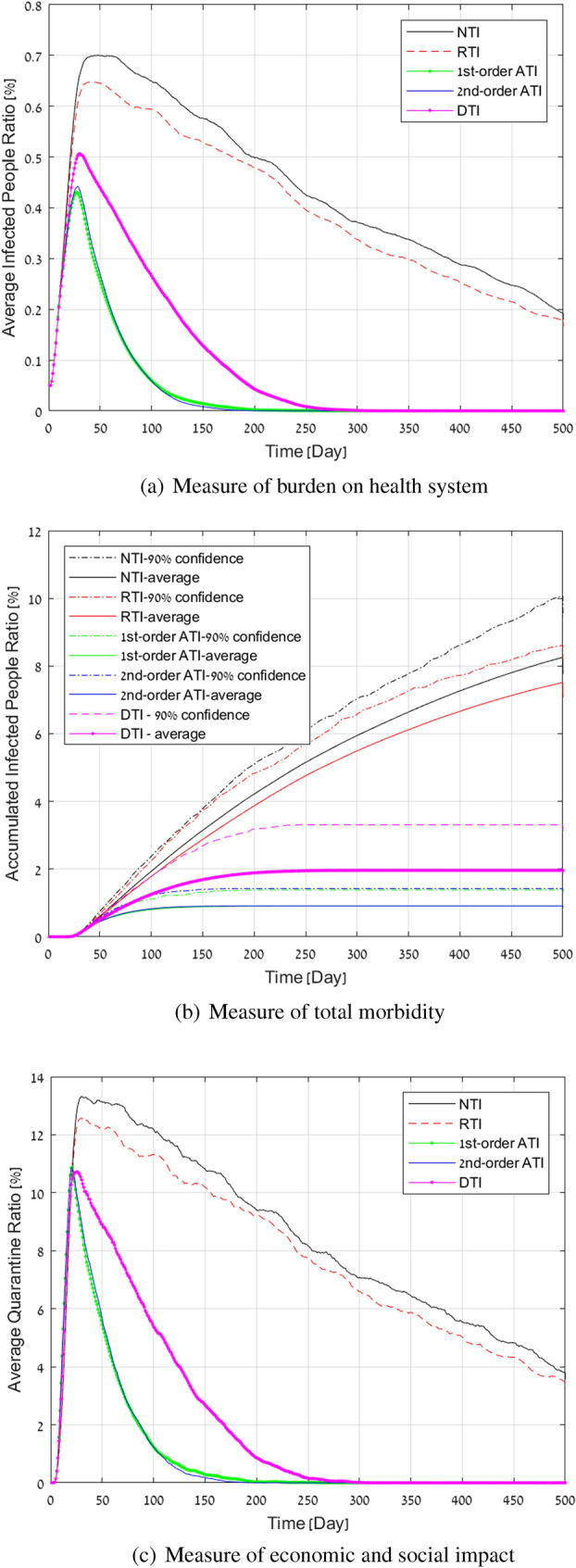
Simulation results for a COVID-19 outbreak in a population of 100,000 people, and testing capacity of 0.3% per day.

**New eruption in a city:** To simulate the case of an early outbreak, we consider the case of 50 infected people in a city of 100,000 people. We also use this case to test the magnitude of success of the quarantine. We assumed that false negative of tests are $10\%$ and changed the quarantine success rate $q_s$ from 0.7 to 1. We assumed that we can test daily $r_T=0.3\%$ of the population. First we present the performance of the various techniques with respect to a quarantine success rate of $70\%$ in Fig. 2.

As shown, as long as the tests are properly controlled by the ATI, the decay time is relatively short even when the quarantine is only observed by $70\%$ percent of the population required to be in quarantine. In contrast,the RTI is practically equivalent to the NTI and both fail to quickly reduce the eruption (because of the low obedience rate). The combination of a low quarantine rate and random testing is incapable of providing an effective means to block the spread, although it does manage to flatten the curve over a very long period. DTI has better performance, yet the active approach is significantly superior in controlling future small outbreaks (DTI requires twice quarantine days, and results in twice morbidity rate). Thus, accurate contact tracing with controlled testing does not require a huge amount of testing to stop the outbreak. We observed (see “Methods” section) that NTI achieves results that are between tens and up to hundreds of percent worse than ATI when the quarantine success rate is between $100\%$ and $70\%$, respectively. Table 2, presents the total infected population, overall quarantine days over the epidemic and the peak infection for all methods for quarantine success rate of $70\%$. It can be seen that the total days lost due to quarantine by NTI is up to 8 times greater than ATI, and even DTI requires up to $100\%$ more quarantine days than ATI, mainly because it does not order people to be tested according to the probability of infection. NTI and RTI also result in 4 times and up to 9 times more infected individuals.

**Table 2:** Performance evaluation for a $70\%$ quarantine success rates.

Measure/method	NTI	RTI	DTI	1st-order ATI	2nd-order ATI
Total $\#$ of infected people	8,256	7,516	1962	909	911
Peak $\#$ of infected people	701	648	507	431	442
Total $\#$ of days in quarantine	4,118,872	3,816,708	1,046,777	509,401	508,265
Curve width of quarantine above $1\%$ (in days)	$>500$	$>500$	244	142	136

**Figure 3:**
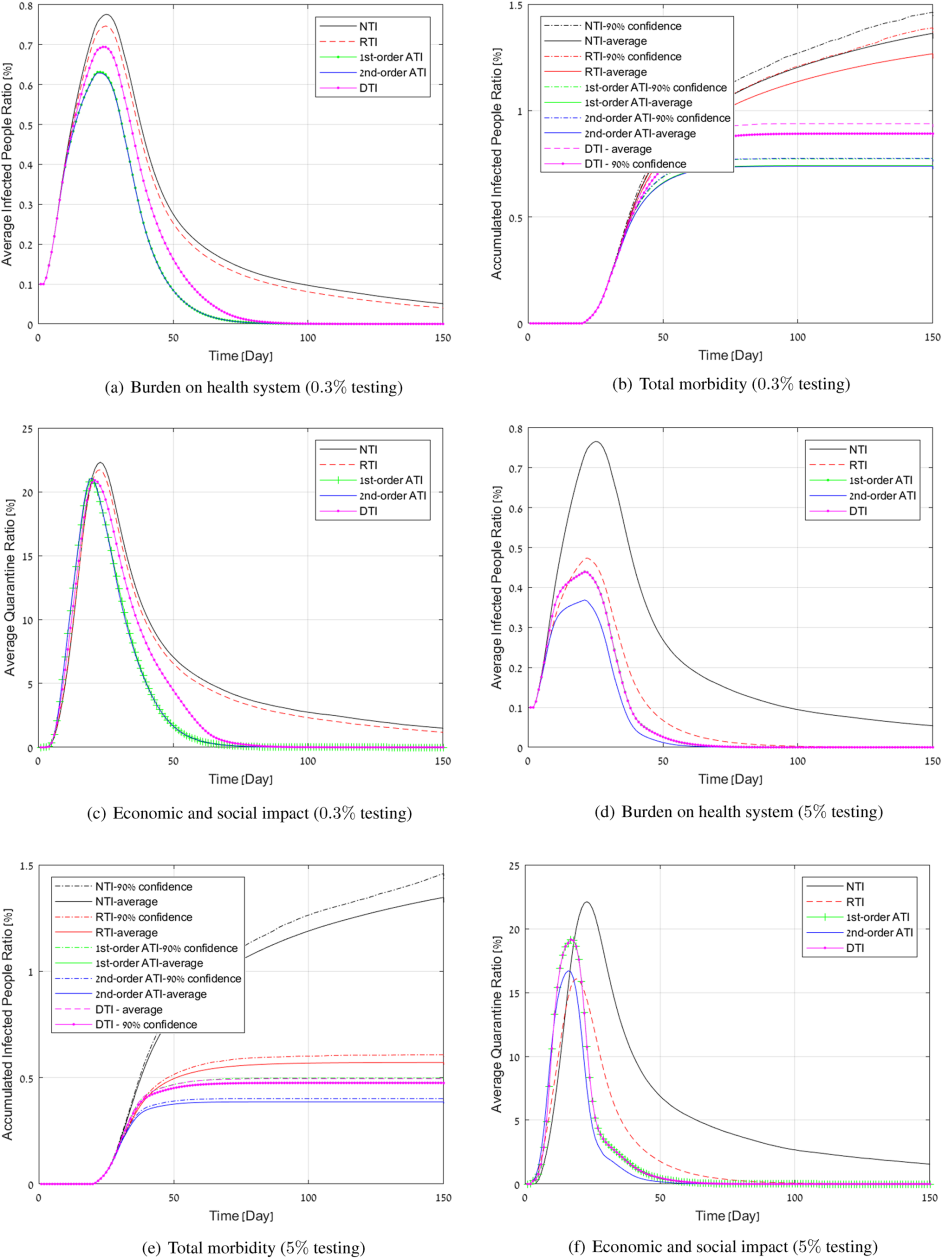
Simulation results for a COVID-19 outbreak in a population of 1 million people. Daily testing capacity ($0.3,5\%$).

**Developed outbreak:** We now present the results of controlling an outbreak with $0.1\%$ of the population already infected for a region of one million people. We assumed a false negative rate of $20\%$ which is typical to current testing methodology. We simulated two scenarios: $0.3\%$ and $5\%$ of population tested daily. In Fig. 3a,d we present the average number of infected people as a function of time. In Fig. 3c,f we present the average number of people in quarantine as a function of time and in Fig. 3b,e we present the total morbidity. For these graphs we also present the upper confidence level of $90\%$ (For other graphs, these are provided in the Supplementary Information). Table 3 provides the accumulated measures for $0.3\%$ daily testing.

The figures show that NTI and RTI performs the worst on all performance measures. To achieve only slightly inferior performance compared to DTI, RTI requires significantly larger amount of testing. DTI is worse than ATI. These results support the observation that a small number of random tests or even based on contact tracing with no prioritization cannot achieve a significant improvement in controlling the spread of COVID-19, while the suggested ATI strategy (both first and second-order) succeeds in doing so. Specifically, the peak load in Fig. 3a under NTI and DTI, respectively, is $25\%$, and $11\%$ higher than ATI. The total morbidity in Fig. 3b under NTI and DTI, respectively, is $85\%$, $20\%$ higher on average. The quarantine curve in the $1\%$ quarantine ratio in Fig. 3c using NTI and DTI, respectively, is $200\%$ and $20\%$ wider than that of ATI. These results demonstrate the importance of controlled testing.

It can be seen that first-order and second-order ATI achieves roughly the same performance for $0.3\%$ testing, whereas ATI2 better exploits the large number of tests. This can be explained by the fact that when the number of tests is relatively small, both strategies give high weight to testing first-order neighbors. Thus, in this case, the first-order approximation is sufficient for effective active testing. In the large number of tests,we see equality of ATI1 and DTI since in this case all first order connections are tested. At this level of tests also the random testing performs quite well. However daily tests of $5\%$ of the population are definitely beyond reach with current means (even in Wuhan at peak around $0.3\%$ of the population has been tested daily.)

**Table 3:** Performance evaluation for a 85% quarantine success rates.

Measure/method	NTI	RTI	DTI	1st-order ATI	2nd-order ATI
Total $\#$ of infected people	13,633	12,673	8917	7420	7384
Peak $\#$ of infected people	7761	7468	6944	6315	6291
Total $\#$ of days in quarantine	8,925,115	8,313,597	5,621,309	4,699,833	4,675,584
Curve width of quarantine above 1% (in days)	$>150$	$>150$	68	51	51

**Figure 4:**
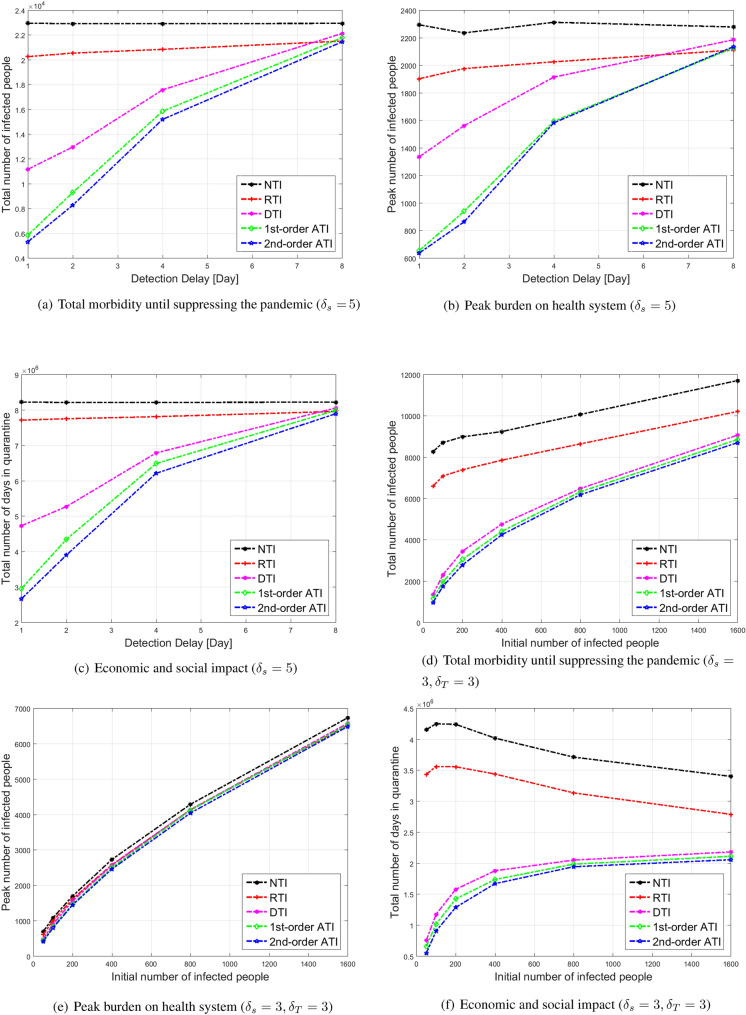
Simulation results for a COVID-19 outbreak in a population of 100,000 people. Daily testing capacity is set to 1%.

**Dependence on parameters:** Next, we test the effects of $\delta _T, \delta _s$ and $r_i$ on the performance. The results demonstrate the importance of taking operational actions quickly to suppress the pandemic spread, in terms of providing test results quickly (i.e., small $\delta _T$), shortening the epidemiological investigation period as well as asking people to enter into quarantine promptly as soon as symptoms appear (i.e., small $\delta _s$). We also show that identifying and responding quickly to outbreaks in early stages (i.e., small $r_i$) is also very important. First, we consider the case of 50 infected people in a region of 100, 000 people. We set the daily test rate to $r_T=1\%$ of the population, as this is the testing rate in many counties today^48^. We present here the results for $\delta _s=5$. Results for $\delta _s=1, 3$ are provided in the Supplementary Information. In Fig. 4a–c, we present the total number of infected people, peak number of infected people, and total number of days in quarantine as a function of $\delta _T$. It can be seen that ATI2 outperforms all other methods in all measures. For $\delta _T=1$ day, the total morbidity in Fig. 4a under RTI and DTI, respectively, is $260\%$, and $105\%$ higher than ATI2, the peak number of infected people in Fig. 4b under RTI and DTI, respectively, is $190\%$, and $105\%$ higher than ATI2. The total number of days in quarantine in Fig. 4c under RTI and DTI, respectively, is $190\%$, $77\%$ higher than ATI2. A significant performance gain of ATI over other methods is observed up to $\delta _T=4$ days. Nevertheless, the performance gain decreases with $\delta _T$, and diminishes for $\delta _T=8$. These results demonstrate the importance of operating efficient test labs that provide test results quickly.

Finally, we set $\delta _T=3, \delta _s=3$, and tested the results as a function of $r_i$. In Fig. 4d–f, we present the total number of infected people, peak number of infected people, and total number of days in quarantine as a function of the number of initial infected people in a region of $N=100{,}000$ people. It can be seen that ATI2 outperforms all other methods in all measures. For $r_i\cdot N=50$, the total morbidity in Fig. 4d under RTI and DTI, respectively, is $400\%$, and $30\%$ higher than ATI2, the peak number of infected people in Fig. 4e under RTI and DTI, respectively, is $40\%$, and $10\%$ higher than ATI2. The total number of days in quarantine in Fig. 4f under RTI and DTI, respectively, is $550\%$, $40\%$ higher than ATI2. A significant performance gain of ATI over other methods is observed up to $r_i\cdot N=800$. For $r_i\cdot N\ge 800$ the performance gain of ATI over DTI diminishes. This can be explained by the fact that as the initial number of infected people is too large, then most of the resources should be dedicated to tracing the close contacts of the infected people. It also can be seen that the benefit of using tests to suppress the pandemic by all methods decreases with $r_i$. This result is expected since controlling such a developed outbreak requires a much larger number of tests, or applying a large-scale lock-down. Another interesting observation is that in Fig. 4e the difference between the peak number of patients when using various policies is approximately constant. This is expected, since for a given $\delta _T, \delta _s$ the process is Markovian and therefore, its peak evolution is independent of the initial state.

## Discussion

Today, health systems around the world are prioritizing the use of COVID-19 tests for people who have either had direct contact with an infected person or are symptomatic. Some countries, such as South Korea, use extensive resources to test people randomly. However, testing capacity is raely beyond $1\%$ of the population^48^.

Our results clearly demonstrate the importance of contact tracing technology, either by localization, or by contact monitoring using short range wireless communication. $\delta _T$ also includes the time required for conducting detailed epidemiological investigations. Typically, when done by humans, these investigations require up to 3 days. In contrast, electronic-based contact tracing significantly shortens this time. As a consequence, it reduces $\delta _T$. It was clearly shown that the shorter $\delta _T$ is the faster the epidemic is contained. In our opinion, these results provide very strong evidence on the necessity of technological contact tracing. Our results also show that the proper use of contact tracing must be complemented with a proper probabilistic analysis of the contact data (number of contacts, duration, etc.) to guide new tests of infection. The larger the testing capability, the more elaborate the algorithms must be to exploit the additional testing capacity.

It is worth noting that the probability of infection used in the stochastic model can be determined based on the distance and duration of contact. For example, being within 1 m of a COVID-19 patient for more than 15 min is defined as close contact by the WHO, yet, measuring the duration of contacts can provide a more elaborate probabilistic model. Intuitively speaking, decreasing the distance or increasing the duration increases the probability of infection. Therefore, by using more accurate distance and duration determination provided by short range wireless communication we might be able to define or learn a probability distribution from the data for the probability of infection. Such new models for infection probability can be easily incorporated in the stochastic model for controlled testing.

We demonstrated that testing people using the methodology of a controlled experimental design in the spatio-temporal dimensions based on belief approximation exploits the available testing capacity much more efficiently. This results in a significant reduction of the burden on the healthcare system, the morbidity, and the negative economic and societal impact. Even a year into the COVID-19 pandemic, no state is testing daily more than $1.2\%$ of its population. This clearly demonstrates the necessity of the proposed methodology to properly exploit the available resources. As shown for an advanced stage epidemic with $0.1\%$ of the population already infected, the quarantine of large portions of the population (e.g., $>1\%$) can be reduced by over two months compared to random testing and by almost two weeks compared to simple neighbor testing, when up to $0.3\%$ of the population is being tested every day. Similarly, the peak load on the health system can be reduced by $18\%$ compared to testing randomly and by $10\%$ compared to contact tracing with no controlled testing. Since the total morbidity is also lower, on one hand and also correlated with the death rate, many lives can be saved as well. Similarly, it was shown that for early stage spread, using controlled testing can prevent the huge deterioration of the situation before it gets out of hand.

While controlled testing ideas can be applied under standard epidemiological investigations, without technological contact tracing, the delay involved and the inability of scaling to the millions, it clearly supports the use of technological contact tracing.

Another interesting observation is that random testing benefits significantly from very large numbers of tests, but that even testing up to $5\%$ of the population daily still yields inferior results on both the deterministic and controlled testing policies. This underscores the importance of contact tracing and accurate probabilistic modeling.

Finally, some comments are in order regarding other factors. We used a false negative rate of $10-20\%$, but results scale favorably even with a $30\%$ false negative rate. The results presented in the paper assumed $85\%$ quarantine success rate. However, it is clear from the further resutls in the Supplementary Information that the performance is only mildly degraded wit $70\%$ quarantine rate. This shows that the method is robust to real world problems.

The importance of the proposed technique is not only in coping with the first wave of the pandemic, but also monitoring new occurrences of the disease over time since it will significantly reduce the overall quarantine rate and accelerate the containment time in future waves.

## Methods

### Stochastic model for controlled testing

The proposed controlled testing scheme requires an underlying stochastic model to be able to use the current testing results to estimate the likelihood of a person being infected given the test results. We used a microscopic stochastic model which includes every agent in the population as described below. We model the connections between all *N* people in an area (e.g., state, district, city) as an undirected graph, where the people are represented by a set ${\mathcal {N}}=\left\{ a_1, a_2,\ldots , a_N\right\}$ of agents and “close contacts” between people who might cause infection are represented by a set *E* of edges. The existence of an edge $(n,i)\in E$ between persons *n*, and *i* is determined based on potential infections. For example, being within 1 m of a COVID-19 patient for more than 15 min is defined as close contact by the WHO^4^ defines an edge in the graph. By using more accurate distance determination provided by short range wireless communication we might be able to define or learn a probability distribution from the data for the probability of infection. This turns the graph into a weighted graph, with time varying weights. For each agent $a_n$ we define his or her set of first-order neighbors by:

1 $$\begin{aligned} {\mathcal {N}}_1(a_n)\triangleq \left\{ a_i\;:\; (n,i)\in E\right\} , \end{aligned}$$

which represent all close contacts with respect to agent $a_n$. Similarly, we define the second-order neighbors of $a_n$ by:

2 $$\begin{aligned} {\mathcal {N}}_2(a_n)\triangleq \left\{ a_j\;:\; \exists a_i\in {\mathcal {N}}_1(a_n) \;\text{ and }\; (i,j)\in E\right\} , \end{aligned}$$

which represent all close contacts with respect to agent $a_n$’s first-order neighbors. We focus only on first and second neighborhoods of agents. Nevertheless, higher neighborhood orders can be defined similarly. Intuitively, if agent $a_n$ is infected, then the probability of infecting other people over the graph decreases as the neighborhood order increases.

We also allow for external independent information regarding each individual’s probability of being infected based on physical measurements.

Each agent has two local states; namely, a physical state $S_P(a_n)$, and controlled state $s_C(a_n)$. The physical state specifies his or her health condition (healthy $S_H$/infected $S_I$/recovered $S_R$ /dead $S_D$), whereas the controlled state specifies whether the agent is in quarantine ($s_Q$) or free ($s_F$).

The active testing algorithm has prior information regarding each agent’s state described by a probability distribution over the four possible values of the physical states, and knowledge of the control state.

The algorithm observes the physical state of the person, either by testing for COVID-19 or by the emergence of symptoms. This observation of $a_n$ is denoted by $o(a_n)$, which equals one if $a_n$ was confirmed infected. Otherwise, it equals zero. Note that observations might result in errors when inferring the physical states; for example, due to false negatives and false positives in COVID-19 tests. These considerations are taken into account in our simulation environment.

### Infections over the population graph

Contagions between people are probabilistic in the stochastic population graph. The probability that infected agent $a_n$ infects $a_i\in {\mathcal {N}}_1(a_n)$ through path $(n,i)\in E$ is denoted by $p_{(n,i)}$. Typically, $p_{(n,i)}$ is set such that the average number of individuals a patient infects is given by the reproduction rate.

The key in operating efficient population testing to stop the spread of the virus is to test the people most likely to be infected (so we can identify them before symptoms appear). The estimated probability of person $a_n$ being infected is denoted by $p_n$, which we refer to as a *belief*. Let ${\mathcal {G}}$ be the graph topology, including the infection probability rules over the graph. Let ${\mathcal {H}}$ be the history of the revealed knowledge by the algorithm up to the current time, including all controlled states, and observations. Then, at each given time the ATI algorithm updates the beliefs based on ${\mathcal {G}}, {\mathcal {H}}$:

3 $$\begin{aligned} p_n=f_n({\mathcal {G}}, {\mathcal {H}}), \ n=1,\ldots ,N. \end{aligned}$$

**Figure 5:**
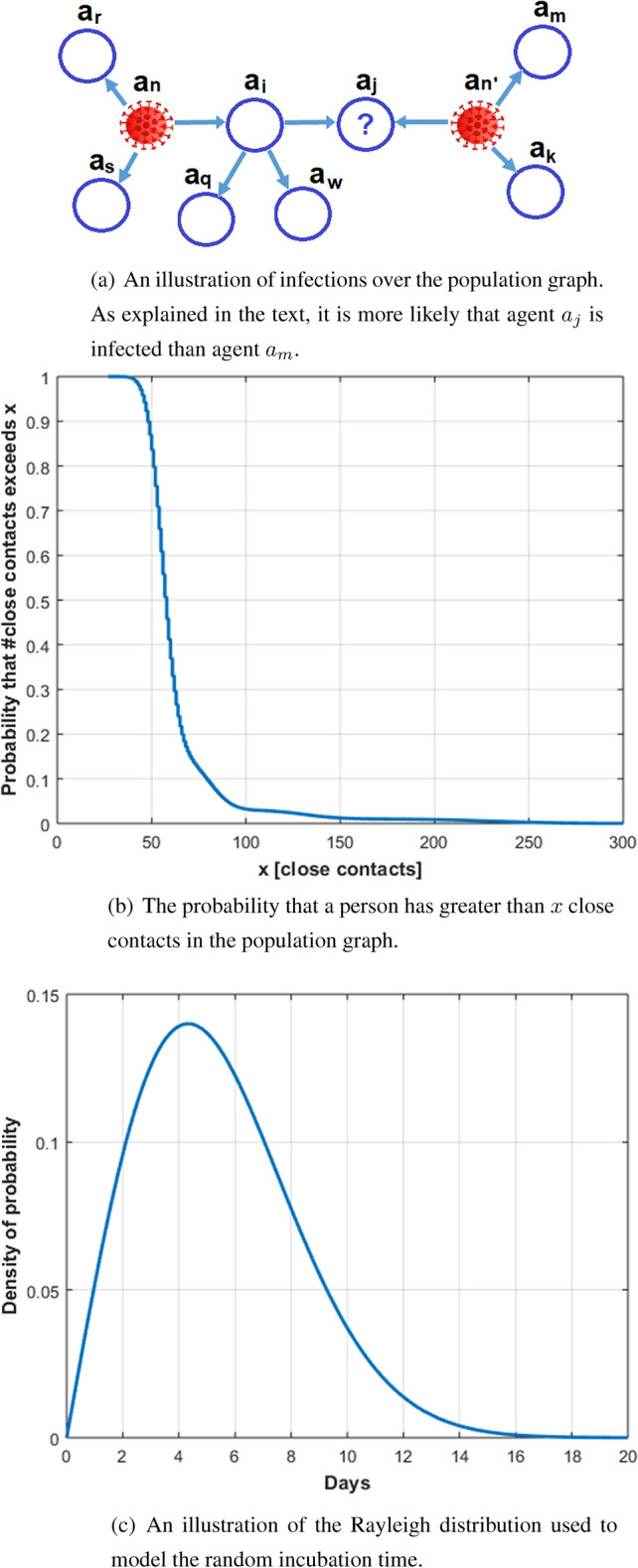
Illustrations for model settings as described in the “Methods” section.

An illustration is provided in Fig. 5a in the end of this section, where the population graph contains two infected patients $a_n$, and $a_{n^{\prime }}$. Given that these patients are identified, we can use the graph topology, and the infection probability over the edges to estimate the probability that $a_j$ is infected. Furthermore, assuming that $a_{n^{\prime }}$ has an equal probability to infect his or her first-order neighbors $a_j, a_m, a_k$, we can further infer that it is more likely that $a_j$ is infected than $a_m$ is (since person $a_j$ is a first-order neighbor of patient $a_{n^{\prime }}$, as well as a second-order neighbor of patient $a_n$, whereas person $a_m$ is a first-order neighbor of patient $a_{n^{\prime }}$, and only a fourth-order neighbor of patient $a_n$), i.e., $p_j>p_m$. Therefore, applying efficient controlled testing strategy should prioritize testing $a_j$ over $a_m$.

### Details of the ATI algorithm

We now provide more detailed explanation on the implementation of the ATI algorithm. The algorithm consists of 4 steps as follows:

1. **Identifying infected people and isolating them:** For the released population, check whether there are new infected people based on symptoms or preceding test results if tested. Send those infected people to quarantine until recovered. Denote these people by $\left\{ a_n\right\} _{n=1}^{A}$.
2. **Isolating first-order neighbors in quarantine:** Order the first-order neighbors $\left\{ {\mathcal {N}}_1(a_n)\right\} _{n=1}^{A}$ into quarantine for at least 14 days. Release them if symptoms have not appeared, or they have recovered, or if test results were negative if tested.
3. **Updating the belief over the population graph:** Update the beliefs $p_j$ for all people in the population graph based on new information (e.g., identifying new infected people, isolating new people).
  Note that the beliefs are updated temporally by taking into account practical aspects of COVID-19 effects on patients. For example, the probability of a second-order neighbor of an identified patient (who was quarantined when detected) being infected decreases with time as long as symptoms do not appear. Therefore, the beliefs must be decreased for all people who are not identified at each time step. Furthermore, the belief of a person that was tested and obtained negative test results should be decreased. This side information is captured by the history ${\mathcal {H}}$. A detailed development of the belief update is given in the next subsections.
4. **Actively testing the most likely infected people:** Test all people with top $N_T$ beliefs among the entire population, regardless of their symptomatic state.
5. **Repeat:** Go back to Step 1 (for instance, a single day ended).

### Development of second-order approximation of the belief

The development of the second-order approximation of the belief used in ATI algorithm is described next. Let

4 $$\begin{aligned} {\mathcal {P}}_1(a_j)=\left\{ (n^{\prime },j):\; j\in {\mathcal {N}}_1(a_{n^{\prime }})\right\} , \end{aligned}$$

and

5 $$\begin{aligned} {\mathcal {P}}_2(a_j)=\left\{ (n,i,j):\; i\in {\mathcal {N}}_1(a_n),\; j\in {\mathcal {N}}_1(a_i)\right\} \end{aligned}$$

be the sets of all one-hop, and two-hop infection paths of person *j*, respectively. Then, the probability that person $a_j$ is infected through infection paths ${\mathcal {P}}_1(a_j)$ or ${\mathcal {P}}_2(a_j)$ only is given by:

6 $$\begin{aligned} p_{j}&= 1-\prod _{\begin{array}{c} (n^{\prime },j)\in {\mathcal {P}}_1(a_j) \end{array}}\left( 1-p_{(n^{\prime },j)}\right) \nonumber \\&\quad \times \prod _{\begin{array}{c} (n,i,j)\in {\mathcal {P}}_2(a_j) \end{array}}\left( 1-p_{(n,i,j)}\right) \nonumber \\&= 1-\prod _{\begin{array}{c} (n^{\prime },j)\in {\mathcal {P}}_1(a_j) \end{array}}\left( 1-p_{(n^{\prime },j)}\right) \nonumber \\&\quad \times \prod _{\begin{array}{c} (n,i,j)\in {\mathcal {P}}_2(a_j) \end{array}}\left( 1-p_{(n,i)}\cdot p_{(i,j)}\right) . \end{aligned}$$

By assuming that $p_{n,i}\ll 1$ for all *n*, *i*, we get:

7 $$\begin{aligned} \displaystyle p_{j}\approx \sum _{\begin{array}{c} (n^{\prime },j)\in {\mathcal {P}}_1(a_j) \end{array}}p_{(n^{\prime },j)} +\sum _{\begin{array}{c} (n,i,j)\in {\mathcal {P}}_2(a_j) \end{array}}p_{(n,i)}\cdot p_{(i,j)}. \end{aligned}$$

We use this approximation to update the beliefs over the population graph in the simulated models described next.

### Using simulated models to validate the ATI policy

#### Description of the graph topology

We simulated a COVID-19 outbreak environment with *N* people. The outbreak is initiated by $N_I$ infected people. In the simulations we set $N=100{,}000$, and $N_I=50$, or $N=1{,}000{,}000$, and $N_I=1000$. The degree distribution of the population graph captures different volume levels to model different types of social connections in the graph. For example, an office worker might have 25 close contacts, while a public service worker might have 200 close contacts. An example of the tail probability of the degrees in the population graph that we used in the simulations is illustrated in Fig. 5b in the end of this section.

#### Simulating symptomatic and asymptomatic people

An infected person can be asymptomatic or symptomatic. Let $r_a$ and $N_a$ be the ratio of asymptomatic people over the entire population, and the number of asymptomatic people, respectively. In the simulations we set $30\%$ of people as asymptomatic (i.e., $N_a=r_a\cdot N=0.3\cdot N$). For asymptomatic people, symptoms do not appear and the only way to verify their condition is by testing them. For symptomatic people, we model the appearance time of symptoms by a Rayleigh distributed random variable with parameter $\sigma =5.1/\sqrt{2\log (2)}$, as illustrated in Fig. 5c. This distribution models the appearance of symptoms mostly between day 2 and 11 from the day the person was infected, with a median of day 5.1, and a probability that symptoms will appear after day 11 of less 0.03. The distribution model and its parameters were chosen to fit the empirical incubation time recently reported in^49^.

#### Transiting to static states

Static states in the simulations represent a state of a person who cannot infect other people or be infected by other people. This state captures a person who was infected and recovered, or sadly a person who was infected and died. The time between the day of infection and transitioning to the static state was set to $D_s=21$ in the simulations.

#### Placing people in quarantine

Once a positive observation $o(a_n)$ for person $a_n$ is obtained, $a_n$ enters quarantine. This is done if symptoms appear for person $a_n$, or if the person was scheduled for testing and a positive test result was obtained. The person enters quarantine until he or she transitions to the static state (recovers or dead), or after the recovery time has elapsed (set to 21 days in simulations) in the case of a false positive (we set 0.01 false positive rate in simulations). In addition, all his or her first-order neighbors ${\mathcal {N}}_1(a_n)$ enter quarantine for at least $D_q$ days, where $D_q=14$ in the simulations, which is the typical isolation time used in many countries. People in quarantine cannot infect other people or be infected by other people. After 14 days, individuals in ${\mathcal {N}}_1(a_n)$ are released if symptoms have not appeared. To further model realistic situations, we assumed that $\delta _s$ days elapse between the day symptoms appear and the day a person and his or her first-order neighbors enter quarantine. We also assumed that $\delta _T$ days elapse between the day the test is performed and the result is received, and consequently the person and his or her first-order neighbors enter quarantine if positive.

#### False negatives in test results

Efficient COVID-19 tests should have low false negative rates to avoid releasing infected people and infecting other people. Today, COVID-19 tests suffer from roughly $0.1-0.2$ false negative rates. To model this effect, we incorporated a parameter *FN* in the simulation environment. In the simulations, we set *FN* to 0.2.

#### Stochastic infections over the population graph

We set the infection probability oevr the graph by:

8 $$\begin{aligned} p_{(n,i)}=\frac{r_{(n,i)} R_0}{|{\mathcal {N}}_1(a_{n})|}, \end{aligned}$$

where $\sum _{i: i\in {\mathcal {N}}_1(a_{n})}r_{(n,i)}=|{\mathcal {N}}_1(a_{n})|$, where the reproduction rate was set to $R_0=2.3$ fixed for all the individuals in the simulations. This models the case where all people infect the same number of people $R_0$ on average. It models the situation where people with a high degree of infection interacts with each person for less time, or use protective measures that reduce the infection risk (e.g., public service workers). Nevertheless, the simulator is generic and can be updated to simulate different infection probabilities for different people. The constants $r_{(n,i)}$ model the situation where the infection probability is not uniform among first-order neighbors. For example, being within 1 m of a COVID-19 patient for a longer period of time increases the infection probability. In the simulations, we set $r_{(n,i)}$ to 0.25 for half of the first-order neighbors, and 1.75 for the other half, for each person.

#### Updating the belief in ATI algorithm

We simulated the suggested ATI algorithm using first-order and second-order approximations of the beliefs in Eq. (6), respectively, i.e.,

9 $$\begin{aligned} p_{j}\approx \sum _{\begin{array}{c} (n^{\prime },j)\in {\mathcal {P}}_1(a_j) \end{array}}\frac{r_{(n^{\prime },j )}R_0}{|{\mathcal {N}}_1(a_{n^{\prime }})|} \end{aligned}$$

under the first-order ATI, and

10 $$\begin{aligned} p_{j}\approx \sum \limits _{\begin{array}{c} (n^{\prime },j)\in {\mathcal {P}}_1(a_j) \end{array}} \frac{r_{(n^{\prime },j)}R_0}{|{\mathcal {N}}_1(a_{n^{\prime }})|} +\sum _{\begin{array}{c} (n,i,j)\in {\mathcal {P}}_2(a_j) \end{array}}\frac{r_{(n,i)} r_{(i,j)}R_0^2}{|{\mathcal {N}}_1(a_n)|\cdot |{\mathcal {N}}_1(a_i)|} \end{aligned}$$

under the second-order ATI.

In addition, we used temporal updates of the belief. We reduced the belief of all people by multiplying it by a forgetting factor $\alpha$ at each iteration. This gives higher weight to recent observations. Specifically, at each iteration, we update the belief by:

11 $$\begin{aligned} p_n\leftarrow \alpha \cdot p_n, \end{aligned}$$

where $\leftarrow$ denotes assignment notation. A forgetting factor of $\alpha =0.75$ demonstrated good performance. This fits a residual belief of approximatey $3\%$ after 11 days, which fits the tail of the occurrence symptoms distribution.

Similarly, a negative test result should reduce the belief significantly. The proper way to do this is using a Bayesian update taking into account the false negative rate in the infected population, as well as the overall infection rate in the population. We found that this can be replaced quite well by a forgetting factor of $\beta =0.25$ for a false negative rate of $0.1{-}0.2$ without significant loss. Specifically, at each time a person *n* is tested and the result is negative, we update the belief by:

12 $$\begin{aligned} p_n\leftarrow \beta \cdot p_n. \end{aligned}$$

As usually done in adaptive algorithms, these parameters should be tuned, given the parameters of the environment.

## Supplementary Information

Supplementary Information.
