# Supplementary material for: Suppressing the impact of the COVID-19 pandemic using controlled testing and isolation
Source: Sci Rep. 2021 Mar 18;11:6279. doi: 10.1038/s41598-021-85458-1 (PMC7973790; doi:10.1038/s41598-021-85458-1)
Supplement: Supplementary file 1 — Supplementary Information. [file 41598_2021_85458_MOESM1_ESM.pdf]

Suppressing the impact of the COVID-19 pandemic using controlled testing and isolation

Kobi Cohen[1], Amir Leshem[2*]

[1]School of Electrical and Computer Engineering,

Ben-Gurion University of the Negev, Beer Sheva, Israel

[2]Faculty of Engineering, Bar-Ilan University, Ramat Gan, Israel

[*]Corrresponding author: Amir Leshem; E-mail: amir.leshem@biu.ac.il.

Supplementary information

Additional data regarding the impact of quarantine efficiency on suppressing COVID-19: In this supplementary information we report the simulation results in addition to the results presented in the Performance Evaluation section in the paper. We simulated different values of quarantine efficiency, in which the quarantine was only adhered to $70\%, 80\%, 90\%, 100\%$ percent of the people required to be in quarantine, respectively. As a baseline to our suggested ATI method (first and second order), we simulated the NTI, RTI, and DTI methods, as described in the Performance Evaluation section. The results are presented in Tables 1, 2, and 3. Table 1 shows that the total infected people by NTI is between 1.6 and 9 times higher than ATI. Even using DTI results suffers from up to twice the morbidity as compared to ATI for a 70% quarantine success rate. It can be seen in Table 2 that the peak number of infected people in NTI is up to 1.6 times higher than ATI. Even using DTI results suffers from up to 1.17 times the peak number of infected people as compared to ATI for a 70% quarantine success rate. Finally, in addition to the effectiveness of ATI in terms of reducing the burden on the health system, and reducing the

total morbidity, Table 3 shows that ATI achieves these goals at a relatively small cost in terms of required qurantine days. Specifically, the total days lost for quarantine by NTI are between 1.6 and 8 times more than ATI, and even DTI requires between 1.17 and 2 times more quarantine days than ATI. The average value and upper confidence level of 90% are presented in Figures 1, 2.

In Fig. 3 we present the results for the parametric study in the Results section for $\delta_s = 1, 3$ as a function of δ_T. It can be seen that ATI achieves very strong performance in all measures as compared to other methods for small values of δ_s, δ_T. Furthermore, the performance of all methods that use tests for controlling the pandemic spread decreases as δ_s, δ_T increase. These results demonstrate the importance of taking operational actions quickly to suppress the pandemic spread, in terms of providing test results quickly (i.e., small δ_T), shortening the epidemiological investigation period as well as asking people to enter into quarantine promptly as soon as symptoms appear (i.e., small δ_s).

Table 1: Total # of infected people

Quarantine success/method	NTI	RTI	DTI	1st-order ATI	2nd-order ATI
70%	8,256	7,516	1,962	909	911
80%	2,042	1,658	620	445	432
90%	756	612	385	318	334
100%	434	423	314	266	274

Table 2: Peak # of infected people

Quarantine success/method	NTI	RTI	DTI	1st-order ATI	2nd-order ATI
70%	701	648	507	431	442
80%	509	488	408	349	344
90%	407	374	331	293	308
100%	340	335	295	261	270

Table 3: Total # of days in quarantine

Quarantine success/method	NTI	RTI	DTI	1st-order ATI	2nd-order ATI
70%	4,118,872	3,816,708	1,046,777	509,401	508,265
80%	1,298,703	1,055,431	395,188	286,326	278,950
90%	549,878	446,522	279,297	230,953	242,417
100%	352,256	342,529	255,518	217,295	223,400

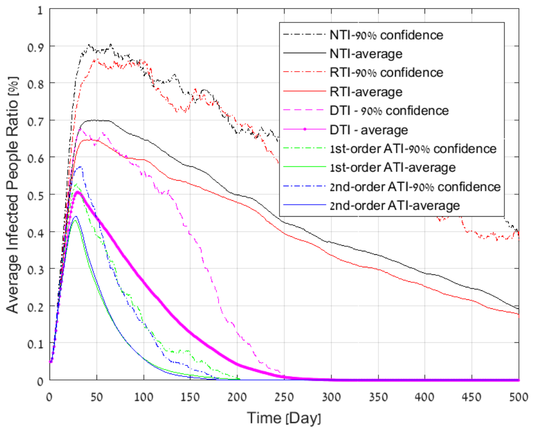

(a) Measure of burden on health system

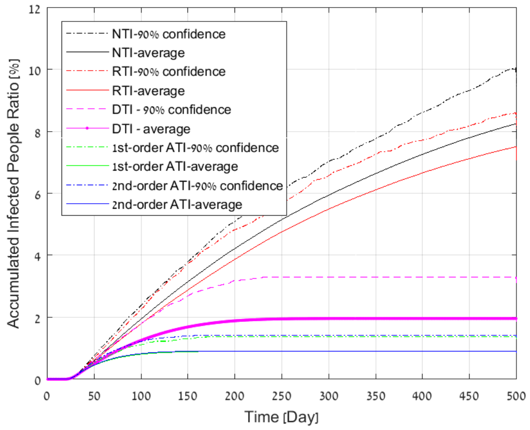

(b) Measure of total morbidity

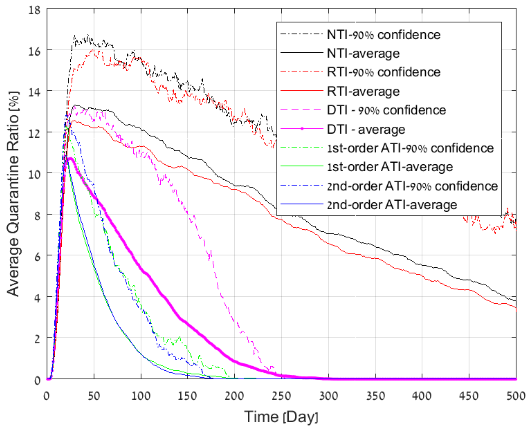

(c) Measure of economic and social impact

Figure 1: Simulation results for a COVID-19 outbreak in a population of 100,000 people, and testing capacity of 0.3% per day. The average and upper confidence level of 90% are presented.

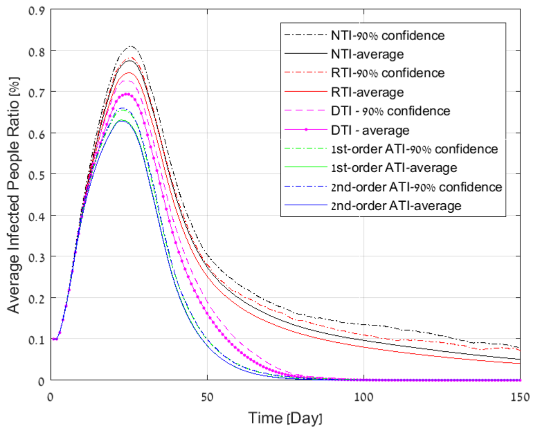

(a) Burden on health system (0.3% tetsing)

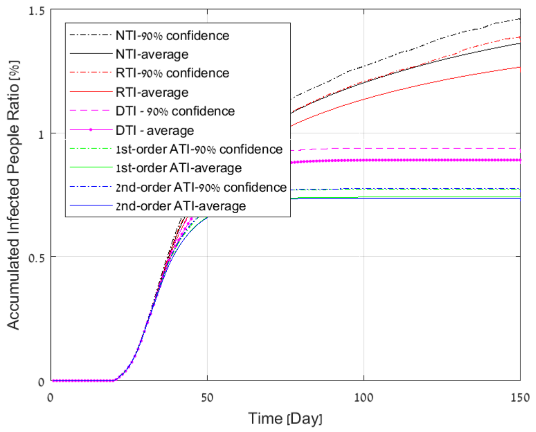

(b) Total morbidity (0.3% tetsing)

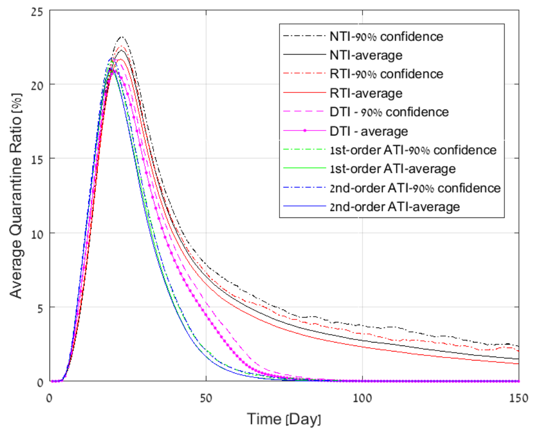

(c) Economic and social impact (0.3% tetsing)

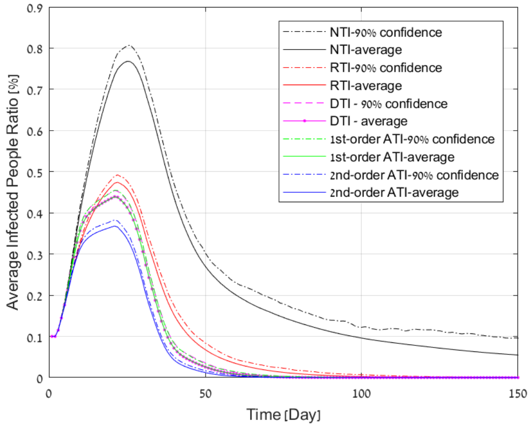

(d) Burden on health system (5% tetsing)

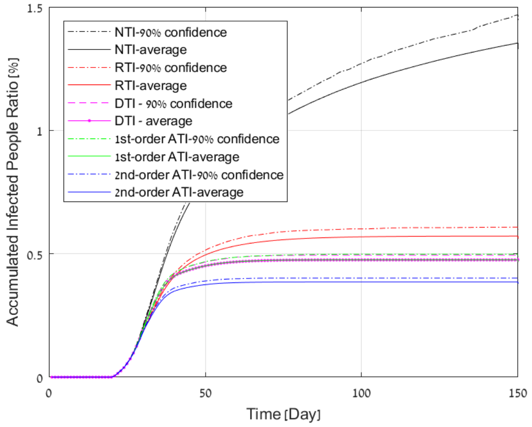

(e) Total morbidity (5% tetsing)

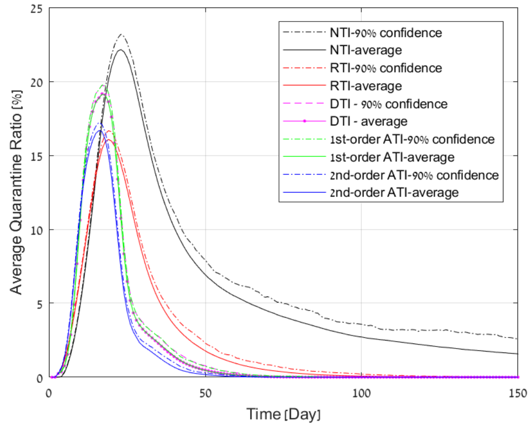

(f) Economic and social impact (5% testing)

Figure 2: Simulation results for a COVID-19 outbreak in a population of 1 million people. Daily testing capacity $(0.3\%, 5\%)$. The average and upper confidence level of 90% are presented.

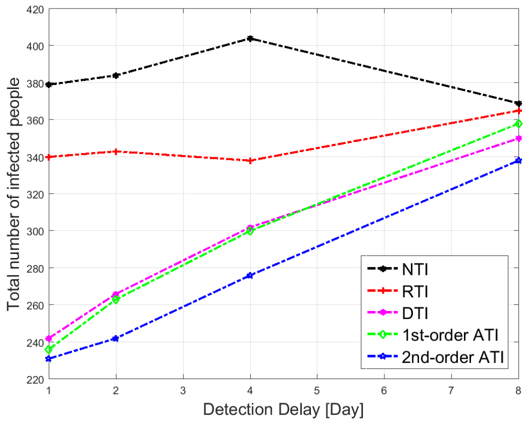

(a) Total morbidity until suppressing the pandemic ($\delta_s = 1$)

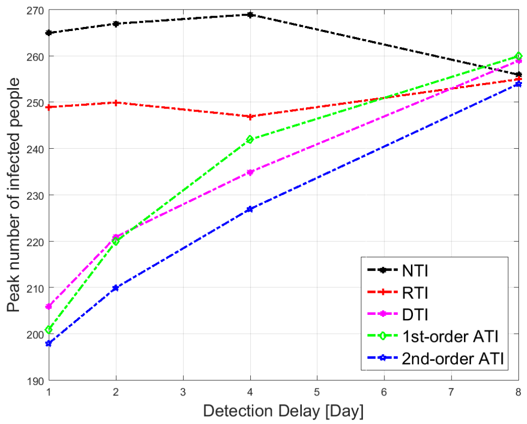

(b) Peak burden on health system ($\delta_s = 1$)

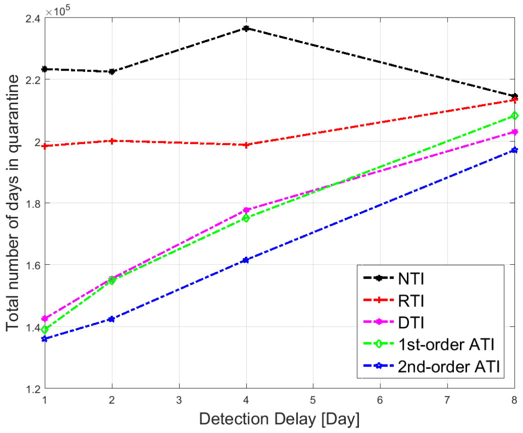

(c) Economic and social impact ($\delta_s = 1$)

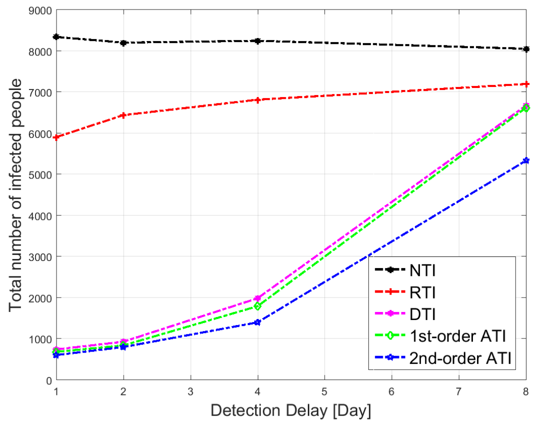

(d) Total morbidity until suppressing the pandemic ($\delta_s = 3$)

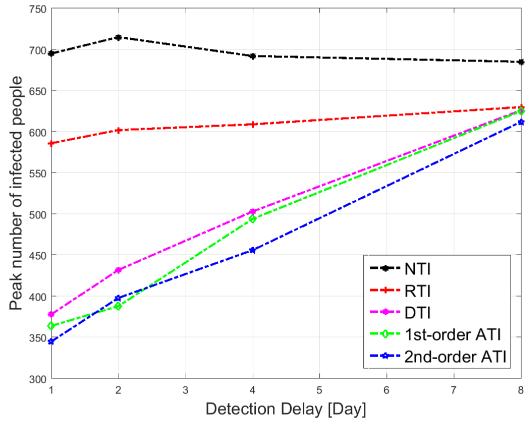

(e) Peak burden on health system ($\delta_s = 3$)

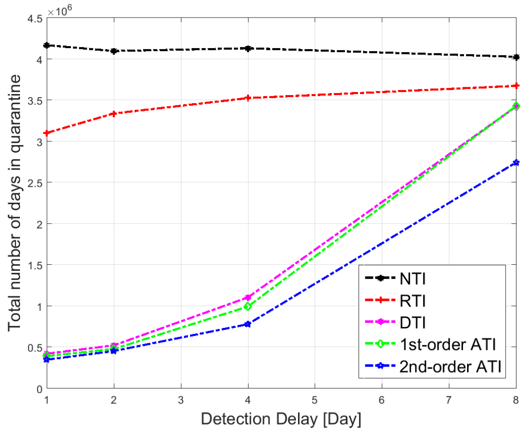

(f) Economic and social impact ($\delta_s = 3$)

Figure 3: Simulation results for a COVID-19 outbreak in a population of $100,000$ people. Daily testing capacity is set to 1%.
